# Nanomaterials and security in occupational and forensic medicine: insights from nanotoxicology

**DOI:** 10.3389/ftox.2024.1476398

**Published:** 2024-11-13

**Authors:** Lang Tran, Michele Treglia, Luca Coppeta, Cristiana Ferrari, Margherita Pallocci, Luisa Campagnolo, Luiz C. De Miranda Junior, Bruno Piccoli, Sharyn Gaskin, Francisco Cortes Fernandes, Fabio Dantas Filho, Pierluigi Passalacqua, Antonio Pietroiusti, Lorenzo Ippoliti, Mario Bragaglia, Francesca Nanni, Andrea Magrini, Luigi Tonino Marsella

**Affiliations:** ^1^ Institute of Occupational Medicine, Edinburgh, United Kingdom; ^2^ Department of Biomedicine and Prevention, Section of Legal Medicine, Social Safety and Forensic Toxicology, University of Rome Tor Vergata, Rome, Italy; ^3^ Occupational Medicine, Department of Biomedicine and Prevention, University of Rome Tor Vergata, Rome, Italy; ^4^ Department of Occupational Medicine, PhD program in Social, Occupational and Medico-legal Sciences, University of Rome Tor Vergata, Rome, Italy; ^5^ Department of Surgical Sciences, PhD program in Applied Medical Surgical Sciences, University of Rome Tor Vergata, Rome, Italy; ^6^ American Better Health Organization 665 Union Blvd Allentown, Allentown, PA, United States; ^7^ Adelaide Exposure Science and Health, School of Public Health, University of Adelaide, Adelaide, SA, Australia; ^8^ National Association of Occupational Medicine (ANAMT), São Paulo, Brazil; ^9^ Department of Occupational Service of Hospital de Clínicas de Porto Alegre - HCPA, Porto Alegre, Brazil; ^10^ Department of Public Health and Infectious Diseases, “Sapienza” University of Rome, Rome, Italy; ^11^ Saint Camillus International University of Health and Medical Sciences (UniCamillus), Rome, Italy; ^12^ Department of Enterprising Engineering “Mario Lucertini”, University of Rome Tor Vergata, Rome, Italy

**Keywords:** nanobombs, occupational medicine, thermobaric, forensic medicine, nanotoxicology (NT)

## Abstract

Nanoenergetics are defined as a class of nanomaterials that possess the ability to release energy in certain situations. These properties have been studied and deepened in recent years, so much so that nanoenergetics have been introduced into the use of the weapons industry, among others. It is therefore an emerging reality that deserves attention with regard to potential harmful effects on human and environmental health. it has been suggested that nanoenergetics may have genotoxic and immunotoxic effects, among others. Problems related to exposure to nanonenergetics can therefore potentially affect both exposed workers (both in the production and use phase) and the civilian population, if used in war scenarios, for example,. Starting from these assumptions, the INNOTOX research project aims to contribute to the in-depth study of the toxicity of nanonenergetics, through an integrated approach involving experts in occupational and forensic medicine, nanotoxicology and bioengineering.

## Introduction

Chemicals are part of our everyday lives. All living and inanimate matter is made up of chemicals, and virtually every manufactured product involves chemicals. Many chemicals, when used correctly, can make a significant contribution to improving our quality of life, health, and wellbeing. However, other chemicals are highly hazardous and, if managed incorrectly, can harm our health and the environment (Prüss-Ustün et al., 2011). In addition, several chemicals which are tested as relatively safe in the micrometric range, may become harmful in the nanometric size (Trache and DeLuca, 2020). Nanoenergetics materials (nEMs) are a class of nanomaterials that exhibit energetic properties, such as the ability to store and release energy in a controlled manner. Considering this perspective, the authors aim to present insights that are considered relevant to the emerging and yet little-explored issue of nanoenergetics and their impact on human and environmental health.

## Discussion

nEMs are composed of nano-sized fuel and oxidizer with or without additives and are investigated for their capability to be sources of high heat release rates and tailored burning rates with a high combustion efficiency (Trache and DeLuca, 2020).

These materials have the potential to revolutionize a wide range of applications, including explosives, propellants, and pyrotechnics. Furthermore, because of their light weight, they are suitable for drone and missile transport over great distance and deliver a considerable blast at relatively modest load.

However, there is growing concern about the potential intentional toxicity of nEMs, as their small size and unique properties may allow them to interact with biological systems in unexpected ways. Despite the increasingly widespread use of these chemicals and the interest they arouse in the scientific community due to their potential usage in the weapons industry, for instance, it must be emphasized that the potential effects on human, animal and environmental health are still poorly understood. This is an issue that is scarcely addressed, but which, considered the widespread use of nEMs, deserves the attention of the scientific community. It has been highlighted that nEMs enter the environment either in the pristine state from spills during production, transport or storage of munitions or may disseminate in their post-combustion state. Due to their size, it has been suggested that aerosolization can be considered as a major transport route and also be adsorbed into the soil or other matrices. Living organisms may be exposed to nanoparticles in different ways, including inhalation of airborne particles (respiratory tract), ingestion (gastrointestinal tract) and dermal contact (skin) (Nancy, 2018).

As mentioned earlier, inhalation appears the major and the most dangerous kind of exposure for both humans and animals.

In the investigation of toxicity related to the exposure to nEMs, it should also be considered that these chemicals may contain metals and metal-based nanoparticles which may lead to the release of metal ions. Regarding thermobaric explosives which increasingly make use of nanoenergetics materials as one of the main ingredients (aka. “nanobombs”). The danger to human health lies not only in the direct traumatic effects linked to the intense release of energy or the nanoparticle exposure to the ocular system or the eye as a whole but also in the possibility that the open wounds caused by the traumatic event may represent a gateway through which the nanoparticles released as a result of the explosion penetrate inside the human organism. Once, the nanoparticles enter the blood stream through the open wound, they are quickly covered by a protein corona which facilitates their entry into body organs (e.g., liver, kidney). At a relatively large secondary organ dose, this can trigger immune toxicity as a form of sepsis but unlike our current medical understanding of sepsis, which is caused by bacterial invasion, this “metal sepsis” cannot be treated with antibiotics.

The potential toxicity of nEMs, either in the short or long term, is thought to arise from several mechanisms, as known in Nanotoxicology, including cellular uptake and accumulation (mainly through specific mechanisms, such as endocytosis or phagocytosis), generation of reactive oxygen species (ROS) that can interact and damage DNA, proteins, and lipids and lead to inflammation and cell death. It has been suggested that nEMs can also interfere with cellular signaling pathways, cell growth and development and the direct damage of cell membranes, leading to leakage of cellular membranes and death (Baker, 2023).

All this considered, some questions arise such as the protective effectiveness of the uniforms currently in use by the military personnel, the provision of environmental protection plans and, above all, the provision of civilians protection and security plans.

To address this issue and to fill this knowledge gap, here we introduce the activities of our research group related to the creation of the Innovative materials and Nanotoxicology for Occupational and Legal Medicine (INNOTOX) research project.

The aim of the INNOTOX project is to further the understanding the toxic mechanisms of nEMs and to mitigate the health and environmental consequences of nEMs exposure, through inhalation, ingestion and dermal routes, by translating our research findings into practical recommendations for occupational health surveillance, preventive measures, and forensic evaluations.

Our group includes experts from a range of disciplines, including occupational medicine, forensic and legal medicine, human pathology, analytical chemistry, and engineers, to address the complex challenges posed by these potentially toxic substances by integrating different areas of expertise.

Our approach is based on the One Health approach, which acknowledges the interdependence of human, 109 animal, and environmental health. From this perspective, the protection of human health passes through the wellbeing of ecosystems. Our aim is to deepen the knowledge about nEMs and especially their intercorrelation with the human, animal, and environmental health.

This approach is crucial for understanding the complex dynamics of nEMs toxicity, considering factors such as bioavailability, exposure routes, species-specific susceptibility, and ecosystem interactions. By adopting a One Health perspective, researchers can gain a more holistic understanding and identify effective solutions that protect both human health and the environment (Machalaba et al., 2021).

The specific objectives of the INNOTOX group are: (i) to identify and quantify nEMs exposure in different settings through advanced analytical techniques and investigate about the consequences of human exposure on tissues and biological fluids (The techniques used will be those currently developed in Nanotoxicology and will be extended as needed). (ii) to help the policymakers in developing and improving evidence-based interventions.

Moreover, in INNOTOX, we actively foster partnerships with national and international research groups, healthcare providers, and regulatory bodies, believing that collaboration is essential to address the multifaceted challenges posed by this new challenge. Thus, INNOTOX hosts an active PhD programme in Social, Legal and Occupational Sciences, training the next-generation of researchers and practitioners to address the complex issues surrounding nEMs exposure. INNOTOX’s work contributes to this goal by providing scientific evidence, developing effective interventions, and fostering international collaboration.

## Data Availability

The original contributions presented in the study are included in the article/supplementary material, further inquiries can be directed to the corresponding author.
